# Application of consecutive polyethylene glycol treatments for modeling the seminal root growth of rice under water stress

**DOI:** 10.1038/s41598-022-06053-6

**Published:** 2022-02-08

**Authors:** Pepi Nur Susilawati, Ryosuke Tajima, Yuti Giamerti, Yi Yang, Muhammad Prama Yufdy, Iskandar Lubis, Koki Homma

**Affiliations:** 1 Graduate School of Agricultural Science, Tohoku University, Sendai, Japan; 2 Assessment Institute for Agricultural Technology of Banten, Serang, Indonesia; 3 Indonesian Center for Horticulture Research and Development, Bogor, Indonesia; 4 Bogor Agricultural University, Bogor, Indonesia

**Keywords:** Drought, Plant morphogenesis

## Abstract

The response of root growth to various osmotic potentials is quite important in assessing the drought resistance of rice. This study modeled seminal root growth by applying consecutive polyethylene glycol (PEG) treatments (from 0 to 25%, 1% step), mathematical equations and noncontact image analysis to quantitatively evaluate the root response. Treatment began after seeds were germinated, and root growth was recorded by a digital camera every day from 7 to 20 days after seeding (DAS). Although the seminal root length (SRL) measured by image analysis slightly varied with DAS, the equations explained the differences in SRL increases under each PEG concentration relatively well (R^2^ = 0.774). The equations also suggested that the maximum seminal root length was observed at 5.9% PEG. This numerical characterization of root growth is an effective means of evaluating drought resistance.

## Introduction

Rice is one of the main food crops worldwide and especially in Asia^1,2^. The demand for rice increases yearly along with the increase in the human population. In 2017, world rice production increased by 0.6%, i.e., 4.5 million tons, for a total of 759.6 million tons (503.9 million tons, milled basis)^3^. However, there are many obstacles to rice production, and one of the current global threats is the vulnerability of rice to environmental stresses such as drought, salinity and extreme temperatures^4,5^. Drought is a constraining factor that has caused a decline in rice productivity worldwide.

Roots are plant organs that play an important role in the supply of water and plant nutrients, which affect plant growth and production under drought. Several studies have shown that one of the root responses to drought in rice is increased root growth and the inhibition of shoot growth^6^. Some studies have also shown that root growth upon rewatering after drought is a key process for production in rice^7,8^. Soil moisture fluctuates with rainfall and evapotranspiration under conditions without standing water, i.e., drought-prone conditions^9^. Evaluation of the root extension ability under various soil moisture concentrations is one of the important factors in analyzing drought resistance.

Drought resistance tests are sometimes carried out by using osmotic solutions^10^. Polyethylene glycol (PEG) is ordinarily used to control osmotic potential to evaluate characteristics of drought resistance^11,12^. Induction of drought in rice using PEG was carried out in a study of the growth responses of traditional Indonesian rice cultivars in the vegetative phase^13^. Several studies have been conducted on the response of root growth under drought conditions using various concentrations of PEG. Adisyahputra et al.^14^ proposed that an 18.1% PEG concentration (equivalent to − 6.24 bar of water potential) can be used to determine resistance to drought stress. Halimursyadah et al.^15^ proposed that 25% PEG can be used for the selection of genotypes of rice hybrids with drought resistance. These results show that it is a difficult challenge to determine the optimum concentration of PEG for the evaluation of drought resistance.

Under these situations, quantifying root growth under various drought stress is strongly recommended. Especially characterization by utilizing mathematical equations has quite effective because it quantitatively provides difference in the responses among cultivars and conditions. The equation also can be utilized to simulate root growth. Although these studies were not for root growth, Hirooka et al.^16^ quantified leaf area growth of several rice cultivars under several conditions by utilizing mathematical equations, and simulated dry matter production and yield^17^. The equations are not depended on the physiological mechanisms but contribute to understand phenomena.

Accordingly, this study tried to model seminal root growth by applying treatments with increasing polyethylene glycol concentrations (1% step) and by applying mathematical equations to analyze the root response. For this purpose, since the growth of seminal root length (SRL) need to be continuously monitored by the noncontact method, image analysis was employed. The implications of the results were discussed in terms of the evaluation of drought resistance and modeling of root growth under fluctuating soil moisture contents to further develop this method.

## Materials and methods

**Figure 1:**
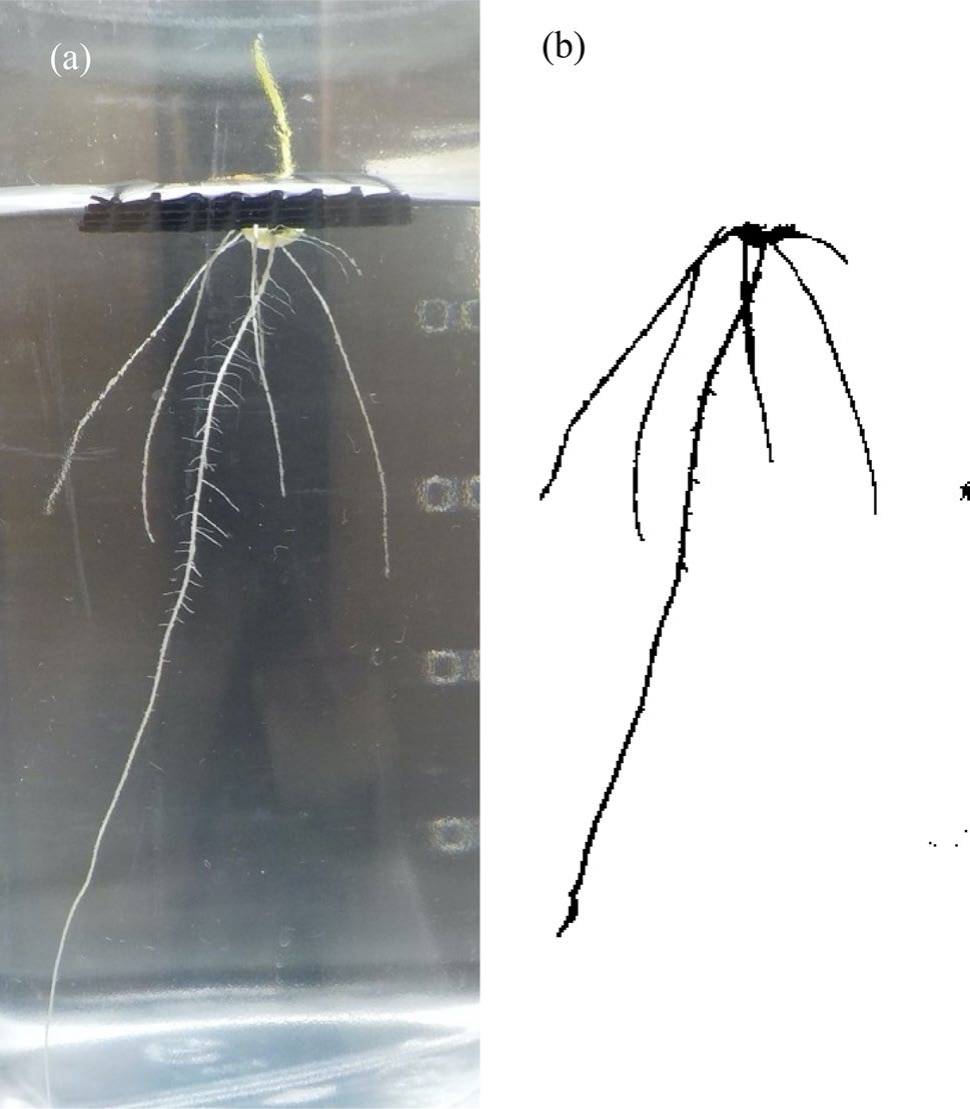
The sample of image analysis. (**a**) Original RGB image and (**b**) its binarized image. The seminal root length in the image was analyzed by the protocol by Tajima and Kato^18^ with ImageJ.

The experiment was conducted in a glasshouse of the Graduate School of Agricultural Science, Tohoku University, Japan, in June 2018. Solutions of PEG (6000 MW, Wako Pure Chemical Industries, Ltd, Osaka) were prepared with consecutive concentrations (1% step) from 0 to 25% dissolved in distilled water. However, observations in 0% PEG and cultivation in 6% PEG concentration failed. Therefore, SRL data at both concentrations were excluded from the analysis. Five hundred milliliters of each solution was made in a transparent bottle (h 150 mm, ϕ 80 mm, vol. 500 ml), and 3 bottles for each PEG concentration were prepared as replications. Rice seeds (Nipponbare) were placed in nonwoven fabric bags, surface-sterilized for 10 min in a solution of 1% HClO (Kao Corporation, Tokyo) and then washed in running water for 1 h. The seeds were soaked in water at 12 °C for 5 days and germinated in darkness at 30 °C for 2 days. The germinated seeds were transplanted to a plastic mesh sheet and placed into the solution in the transparent bottle (one seed for one bottle), which was covered with a fiberboard box and placed in the glasshouse under natural sunlight. Root growth in the bottle was recorded by a digital camera (Coolpix W100, Nikon, Tokyo) every day from 7 to 20 days after seeding (DAS) (for 14 days), consisting of 1008 photos (3 replications × 24 treatments × 14 days). However, since several photos were unsuitable to image analysis, e.x. seminal root grew horizontally to the wall of the bottle, we selected a photo for each treatment for each day. Seminal root length (SRL) was determined as the longest root length by analyzing the images with ImageJ (Fig. 1; Version 1.52e, NIH, https://imagej.nih.gov/ij/^18^). Although the protocol described in Tajima and Kato^18^ also provides an evaluation of total root length, SRL was only evaluated because distinguishing between roots and the wall of bottle was difficult at later DAS. The same setting was used for the image analysis. Rice plants (3 replications for each treatment) were harvested at 30 DAS, and their SRLs were measured directly with a ruler.

The following equation was applied to evaluate SRL (mm) for each PEG concentration (%) as a function of DAS (d).

1 $$SRL=\frac{K}{1-C{\exp}(-r DAS)}$$

2 $$K=a {(PEG+d)}^{b} {\exp}\{-c \left(PEG+d\right)\}$$

where K is assumed to be the full length of the seminal root and C, r, a, b, c and d are regression coefficients. a d^b^ exp (− c d) is y intercept. Equation (1) represents the function for determining the growth of plants^19^. The coefficients were determined by the least square method with R 3.6.2^20^ for the SRL data of all treatments from 7 to 20 DAS analyzed by the image analysis.

### Permissions for collection of seed

Not applicable. Rice cultivar ‘Nipponbare’ is commonly used in experiments in Japan.

### Relevant institutional and national guidelines and legislation

The experimental research followed to the academic disciplines provided by the Association for the Promotion of Research Integrity https://www.aprin.or.jp/en.

## Results and discussion

**Figure 2:**
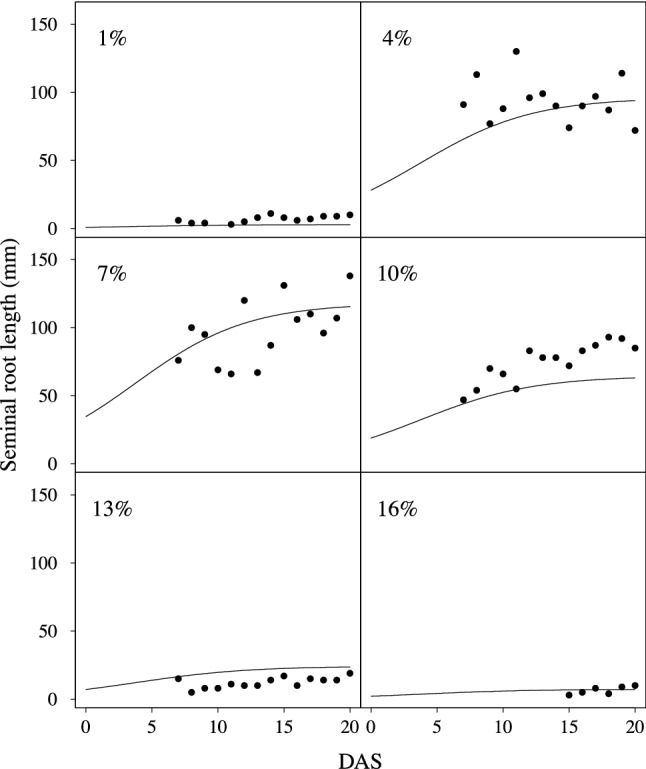
Increases in seminal root length (SRL). Figures in 1% to 3% increments of PEG solution were selected as the examples. Figures in more than 17% PEG solution were omitted because the root growth was strongly suppressed (see Fig. 3). Symbols were measured by image analysis. Lines were estimated by Eqs. (1) and (2).

Although SRL measured by image analysis varied slightly with DAS, Eqs. (1) and (2) relatively well explained differences in SRL increases under each PEG concentration (Fig. 2, R^2^ = 0.774). The original image analysis protocol^18^ was developed for roots destructively collected, but also seemed suitable to roots hydroponically cultivated in a clear bottle. The coefficients C, r, a, b, c and d were estimated as 2.402, 0.235, 5.64, 4.00 0.677 and 0.0, respectively. Figure 2 showed underestimation at 10% and overestimation at 13% PEG concentration. The least square method using all SRL data including all treatments caused the over/under estimation at some PEG concentrations, but the method increased observed numbers and then robustness of parameters.

**Figure 3:**
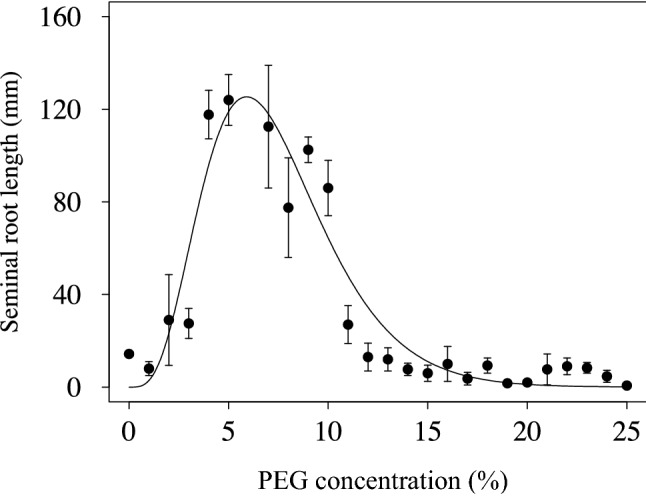
Seminal root length (SRL, symbol) at 30 days after seeding (DAS) and K values estimated by Eq. (2) using data obtained by image analysis (line). SRL was directly measured by a ruler after the plants were harvested.

The assumed full length of the seminal root (K) was estimated by Eq. (2) using the datasets from 0 to 20 DAS measured by image analysis and showed a similarity with SRLs measured directly with a ruler at 30 DAS (Fig. 3). Root mean square error (RMSE) between K and the measured SRL was 13.7 mm. The relatively smaller RMSE also suggests the adaptability of the image analysis protocol^18^ on root growth in a clear bottle. PEG concentrations lower than 3% (equivalent to 0.01 MPa of osmotic pressure^21^) also restricted SRL increases in addition to those greater than 11% (equivalent to 0.15 MPa). Hannan et al.^22^ reported that PEG-induced osmotic stress enhanced new root growth of rice. Under weak osmotic stress conditions, the increase in osmotic potential due to osmoregulation may outweigh the decrease in water potential, resulting in an increase in turgor pressure and accelerated root elongation. Priming effects of PEG were also reported on rice growth under water stress^23^. Oxygen deficiency at germination dynamically alters rate of shoot to root of rice^24^. 30 days hydroponic culture without nutrient in this study might also affect the root elongation. These phenomena might associate root growth suppression under quite low PEG concentration. Since the suppression in this study was rather stronger than those reported in previous studies, further investigation is necessary.

Figure 3 also showed gaps in the SRLs measured at 30 DAS between the 3 and 4% PEG solution treatments and between the 10 and 11% PEG treatments. Inaccuracies in the PEG concentrations might have caused the gaps. However, the solutions were made separately for each bottle, and 3 bottles were prepared as replications, suggesting that the effect of inaccuracy was not large. A smaller concentration step (e.g., a 0.2% step) will be necessary to confirm the responses of seminal root growth to PEG concentrations.

Although the function of PEG in determining the value of K in Eq. (2) needs further validation, the coefficients were determined to explain the experimental SRL data with the least square error. The development of the equation based on the data against PEG is one of the major purposes of this method. From Eq. (2), the following equation was obtained by differentiation:

3 $$\frac{dK}{dPEG}=ab {(PEG+d)}^{b-1} {\exp}\{-c (PEG+d)\}-ac{PEG}^{b} {\exp}\{-c \left(PEG+d\right)\}$$

Equation (3) indicates that K is the maximum at PEG = b/c – d (dK/dPEG = 0). The maximum K was 126 mm at 5.9% PEG (equivalent to 0.04 MPa) in this study. Thus, the numerical characterization of root growth is an effective method for evaluating drought resistance.

Previous studies have shown that soil moisture fluctuations affect root systems, and larger root systems produce more dry matter^25^. Kano-Nakata et al.^26^ reported that soil moisture distribution affects root system architecture. These studies imply that dynamic evaluation of root growth is important. Equations (1) and (2) will be applicable to simulate seminal root growth under fluctuating osmotic pressures that actually occur in the soil under water stress conditions^9^. Since the coefficients were obtained under constant PEG concentrations, although a series of concentrations were prepared, the applicability of this method should first be confirmed by experiments with changing PEG concentrations during seminal root growth.

This study proposed a new experimental design with consecutive PEG treatments. The modeling with mathematical equations based on noncontact image analysis successively evaluated root growth of rice under drought stress mimicked by PEG solution. The consecutive treatments also contribute to enhance repeatability. For example, root growth restriction under low PEG was confirmed by 0 to 4% PEG treatments in this study. Although further validations are necessary to confirm the mathematical equations, the concept of experimental design would be applicable to other stress condition such as salinity stress or nutrient stress. Progress of plant research under stress environment is expected.

## Data Availability

The datasets generated during the current study are available from the corresponding author on reasonable request.
